# COMMD3 Regulates Copper Metabolism via the ATOX1-ATP7A-LOX Axis to Promote Multiple Myeloma Progression

**DOI:** 10.3390/biomedicines13020351

**Published:** 2025-02-04

**Authors:** Yajun Wang, Bo Zhang, Fengjuan Fan, Fei Zhao, Jian Xu, Yuhuan Zheng, Chunyan Sun, Yu Hu

**Affiliations:** 1 Institute of Hematology, Union Hospital, Tongji Medical College, Huazhong University of Science and Technology, Wuhan 430022, China; junjunolivia@126.com (Y.W.); zhangbo19871987@126.com (B.Z.); fengjuan_fan@hust.edu.cn (F.F.); gracezhao911@126.com (F.Z.); xujian01222@163.com (J.X.); 2 Collaborative Innovation Center of Hematology, Huazhong University of Science and Technology, Wuhan 430022, China; 3 Department of Hematology, Institute of Hematology, West China Hospital, Sichuan University, Chengdu 610041, China; yuhuan_zheng@163.com

**Keywords:** multiple myeloma, extramedullary myeloma, COMMD3, copper metabolism

## Abstract

Background: Multiple myeloma (MM) is a hematologic malignancy characterized by the clonal proliferation of plasma cells, with extramedullary myeloma (EMM) being an aggressive form involving malignant infiltration beyond the bone marrow. Copper metabolism is essential for tumor proliferation and metastasis, with copper metabolism MURR1 domain (COMMD) proteins regulating these processes and maintaining copper homeostasis. Dysregulated copper homeostasis contributes to cancer progression, including MM, with elevated copper levels linked to disease aggressiveness and poor prognosis. This study investigates the role of the COMMD3 in mediating MM cell progression, particularly its influence on copper metabolism. Methods: Comprehensive bioinformatics analyses were conducted on bone marrow and extramedullary samples to determine the expression of COMMD3, which was validated through in vitro and in vivo functional assays. The MM cell lines RPMI8226 and MM1S underwent lentiviral transfection for COMMD3 overexpression and knockdown. RNA sequencing was conducted on COMMD3 knockdown cells to identify differentially expressed genes. Functional assays measured cell proliferation, migration, apoptosis, and copper metabolism, with a non-obese diabetic severe combined immune-deficiency gamma (NSG) mouse xenograft model providing in vivo validation. Results: Elevated COMMD3 expression was correlated with extramedullary myeloma and poor prognosis in MM patients. COMMD3 promoted MM cell proliferation and migration, modulating intracellular copper levels, likely through the ATOX1-ATP7A-LOX copper-metabolism-related pathway. High ATOX1 expression was correlated with worse outcomes, and ATOX1 inhibition abolished COMMD3’s effects. Conclusions: This study highlights the pivotal role of COMMD3 in MM progression, particularly via the ATOX1-ATP7A-LOX axis. These findings provide insights into EMM mechanisms and position COMMD3 as a potential therapeutic target. Future research is needed to validate these findings in larger clinical cohorts and to unravel the precise molecular interactions between COMMD3 and copper metabolism proteins.

## 1. Introduction

Multiple myeloma (MM) is the second most prevalent hematologic malignancy, accounting for approximately 1% of all cancers and 10–15% of hematologic malignancies, with an incidence of approximately 1.9 per 100,000 individuals [1]. It is characterized by the clonal proliferation of malignant plasma cells and the aberrant secretion of immunoglobulin [2]. Despite significant advancements in treatment, MM remains incurable, primarily due to the persistence of a dominant clonal population accompanied by diverse subclones [3]. Some of these subclones possess the ability to migrate independently of the bone marrow microenvironment, a process facilitated by disruptions to this niche and downregulation of adhesion molecules [4,5]. These alterations enable extramedullary infiltration into organs such as the skin, lymph nodes, soft tissues, central nervous system, and thoracoabdominal organs [6,7]. This condition, termed extramedullary multiple myeloma (EMM), is linked to a dismal prognosis, with a median survival of approximately 36 months [8,9]. Understanding the molecular mechanisms governing extramedullary infiltration is thus crucial for developing novel therapeutic strategies. However, current insights into the molecular pathways implicated in EMM are sparse, which hampers the development of effective interventions.

The process of EMM likely involves multiple complex pathways, including aberrant signaling in cytokine and chemokine networks and metabolic reprogramming [10]. Emerging evidence suggests that copper metabolism plays a critical role in cancer progression, particularly in regulating cellular migration, invasion, and metastasis [11,12]. Key copper metal chaperones, such as Antioxidant Protein 1 (ATOX1), promote cell migration in various cancers, including breast cancer [13]. ATOX1 mediates its effects by shuttling copper to copper-transporting P-type ATPase (ATP7A), a protein essential for copper transport [14] and enzymatic activity [15]. ATP7A depletion reduces lysyl oxidase (LOX) activity, which in turn limits the metastasis in murine models of Lewis lung carcinoma cells [16]. These findings underscore the importance of copper metabolism in modulating the tumor microenvironment and influencing metastatic behavior. In multiple myeloma, elevated copper ion levels [17] and the pro-apoptotic effects of copper chelation [18] underscore the potential relevance of copper in modulating MM progression. However, there is still a lack of research directly linking copper metabolism to the progression of extramedullary multiple myeloma (EMM), which limits our understanding of copper’s specific role in EMM progression. This raises the need to examine copper-associated molecular pathways in MM.

The copper metabolism MURR1 domain (COMMD) protein family, consisting of 10 highly conserved members [19], has been implicated in diverse biological processes, including tumor progression, metastasis, and copper homeostasis [20,21,22]. These proteins share a conserved COMM domain, which enables protein–protein interactions and facilitates their diverse functional roles [23,24]. COMMD3, a member of this family, has garnered attention for its regulatory role in B-cell migration and humoral immune responses through its interaction with chemokine receptors [25]. Furthermore, COMMD3 inhibitors are being explored as potential therapeutic agents in metastatic prostate cancer [26]. Recent evidence also links COMMD3 to copper metabolism via its interaction with ATP7A, suggesting a potential regulatory axis that may influence cell migration [27]. Despite these findings, COMMD3’s role in MM metastasis remains to be elucidated, prompting further investigation.

This study aims to elucidate the role of COMMD3 in MM cell progression and copper metabolism by addressing the following research question: Does COMMD3 contribute to MM progression and metastasis through the regulation of copper-metabolism-related pathways? This study utilizes a combination of bioinformatic analysis and experimental validation in vitro to uncover potential mechanisms underlying the heterogeneity of extramedullary multiple myeloma and to propose novel molecular pathways contributing to MM metastasis.

## 2. Materials and Methods

### 2.1. Cell Lines

The human MM cell lines RPMI8226, MM1S, MM1R, U266, and NCL-H929 were purchased from the American Type Culture Collection (Manassas, VA, USA). All the cell lines tested negative for mycoplasma (Mycoplasma Real-time qPCR Detection Kit, Yeasen Biotechnology, Shanghai, China) and were authenticated by short tandem repeat (STR) genotyping. The cell lines were cultured in RPMI-1640 (Gibco, Waltham, MA, USA) supplemented with 10% fetal bovine serum (FBS) in a 5% CO_2_ incubator at 37 °C. Only cells in the logarithmic growth phase were selected for experiments, with passage numbers ranging from 5 to 8.

### 2.2. Primary CD138^+^ Cell Isolation

A total of 9 bone marrow aspirates (BM) and 9 extramedullary samples (EM) (pleural effusion, ascites, or tissue homogenates) were collected from 12 MM patients recruited at Union Hospital (Wuhan, China) between September 2023 and October 2024. The diagnosis of MM was confirmed based on the International Myeloma Working Group criteria. Primary MM cells were isolated from bone marrow aspirates or pleural effusion, ascites, or tissue homogenates. Cells were purified using CD138 microbeads according to the manufacturer’s instructions (Miltenyi Biotec, Bergisch Gladbach, Germany).

### 2.3. Antibodies and Reagents

The antibodies and dilution information used for Western blotting are as follows: COMMD3 (Proteintech, Wuhan, China, 26240-1-AP, 1:1000), ATOX1 (Proteintech, Wuhan, China, 22641-1-AP, 1:1000), LOX (Proteintech, Wuhan, China, 17958-1-AP, 1:1000), ATP7A (Santa Cruz, TX, USA, sc-376467, 1:1000), E-cadherin (Wanleibio, Shenyang, China, WL01482, 1:1000), *N*-cadherin (Proteintech, Wuhan, China, 22018-1-AP, 1:1000), β-ACTIN (Antgene, Wuhan, China, ANT321, 1:2000), Goat anti-rabbit or anti-mouse immunoglobulin G (IgG), and horseradish-peroxidase conjugated secondary antibody (Antgene, Wuhan, China, ANT020 or ANT019, 1:5000). The ATOX1 inhibitor DCAC50 was purchased from MedChemexpress, Monmouth Junction, NJ, USA. CuSO_4_ was purchased from Sigma Aldrich, St. Louis, MO, USA.

### 2.4. Lentiviral Transfection

We generated stable COMMD3 knockdown (shCOMMD3 with shNC as the negative control) and overexpression (OE-COMMD3 with OE-NC as the negative control) MM cell lines through lentivirus-mediated transfection. The lentiviral constructs were designed and provided by Genechem (Shanghai, China). MM cells were harvested in the logarithmic growth phase and counted to adjust the density to 1 × 10^5^ cells/mL. The appropriate volume of lentiviral particles was calculated based on the multiplicity of infection (MOI = 80) and viral titer using the following formula: virus volume = (MOI × number of cells)/viral titer. HiTransG P transfection reagent was included in the transduction mixture. Cells were incubated at 37 °C in a 5% CO_2_ incubator for 12 h. Cell health was monitored, and the medium was changed as required. Seventy-two hours post-infection, 1 μg/mL puromycin was added to the culture medium to select for successfully transduced cells. The shRNA sequence used for targeting *COMMD3* is GCAGCATGGAACAATTACA.

### 2.5. CCK-8 Assay

Treated cells were seeded into a 96-well plate at a density of 8 × 10^3^ to 1 × 10^4^ cells per well and incubated for 24–72 h as required. To assess the effect of Cu on cell proliferation, varying concentrations of CuSO_4_ (0.1 μM, 0.5 μM, 1 μM, and 5 μM) were added to the culture medium, and cells were incubated for 24 to 48 h as required. On the day of analysis, 10 μL of CCK-8 reagent was added to each well, followed by incubation at 37 °C in the dark for 1 to 4 h. Absorbance at 450 nm was measured using a Multiskan™ GO microplate spectrophotometer (Thermo Fisher Scientific, Gillingham, UK).

### 2.6. Transwell Migration Assay

Treated cells were seeded at a density of 3 × 10^5^ cells per well into the upper chamber of a 24-well Corning Costar insert with an 8 μm pore size, using 200 μL of FBS-free medium. The lower chamber was filled with 600 μL of complete medium containing 20% FBS. To assess the effect of Cu on cell migration, varying concentrations of CuSO_4_ were added to the chamber, and cells were incubated for 12 to 24 h as required. On the day of staining, inserts were removed, and the medium was carefully aspirated. Cells were fixed with methanol for 25 min, followed by three washes with PBS. Crystal violet was used to stain the cells for 25 min, after which the inserts were washed three times with PBS and air-dried. Any remaining cells on the upper side of the insert were gently removed with a cotton swab. Cells on the lower surface were visualized and photographed using an inverted microscope, and the data were analyzed using ImageJ 1.43 software.

### 2.7. RNA Isolation and qRT-PCR

Total RNA was extracted using RNAiso Plus Reagent (Takara, Shiga, Japan). After phase separation with chloroform and centrifugation at 12,000× *g* for 15 min at 4 °C, the aqueous phase was mixed with isopropanol to precipitate RNA. The pellet was washed with 75% ethanol, air-dried, and resuspended in 20–50 µL of RNase-free water. RNA was stored at −70 °C or used for downstream applications. The reverse transcription reaction was set up according to the PrimeScript™ RT Master Mix kit (Takara, Kyoto, Japan) instructions and performed in a PCR machine. qPCR reactions were prepared using a TB Green^®^ PrimeScript™ RT-PCR Kit (Takara, Kyoto, Japan). The qPCR was run with the specified cycling conditions, followed by a melting curve analysis to check the specificity of the products. The relative mRNA levels were calculated using GAPDH as the housekeeping gene using the 2^−ΔΔCt^ method. The primers used to assess the expression levels of the genes *COMMD3*, *ATOX1*, *ATP7A*, and *LOX* are listed in Supplementary Table S1.

### 2.8. Western Blot

Proteins were extracted with cell lysis buffer, supplemented with protease inhibitors, and lysed on ice for 30 min. Protein concentrations were quantified using the bicinchoninic acid (BCA) assay (Thermo Fisher Scientific, Waltham, MA, USA). For Western blot, samples were separated with sodium dodecyl-sulfate polyacrylamide gel electrophoresis (SDS-PAGE) and transferred from gel to PVDF membranes. After being blocked by skim milk, the PVDF membranes were incubated with corresponding primary antibodies. Then, the membranes were incubated with corresponding Goat Anti-rabbit (mouse) IgG. The proteins were finally visualized and recorded by the ChemiDocTM MP imaging system (Bio-Rad, Hercules, CA, USA). β-actin served as the loading control.

### 2.9. Copper Detection

The concentration of copper (Cu) was measured using a Copper (Cu^2^⁺) Colorimetric Assay Kit (Elabscience, Wuhan, China), following the instructions. For the detection of Cu in the indicated MM cells, 2 × 10^6^ cells were lysed in 0.15 mL of lysis buffer, incubated on ice, and centrifuged to collect the supernatant for copper. The copper concentration was calculated by a determination of optical density at a wavelength of 580 nm.

For the inductively coupled plasma mass spectrometry (ICP-MS) analysis, 1 × 10^7^ cells per group were collected, washed twice with PBS, and the cell pellets were submitted for ICP-MS analysis. All values were acquired in triplicate for each sample.

### 2.10. Flow Cytometry for Apoptosis Detection

The cell apoptosis assay was determined according to an Annexin V-FITC/PI apoptosis kit (Becton, Dickinson and Company, Franklin Lakes, NJ, USA). A total of 1 × 10^6^ cells was collected and washed thoroughly with PBS. The cells were then resuspended in 200 μL of 1× Binding Buffer. Each sample was stained with 5 μL of PI Annexin V and 10 μL of Annexin V-FITC. Single-stain controls were prepared by adding either 5 μL of PI or 10 μL of Annexin V-FITC to separate the control samples. The samples were incubated at room temperature in the dark for 15 min. Following incubation, 200 μL of 1× Binding Buffer was added to each tube. The stained samples were analyzed using a BD flow cytometer, and the data were processed with FlowJo v10 software.

### 2.11. Mouse Subcutaneous Tumor Model

The mice were purchased from Cavens Laboratory Animal Co., Ltd., Changzhou, China. Four-week-old female NSG immunodeficient mice, weighing 14–16 g, were acclimated for two weeks in an isolator of the barrier system. On the day of injection, transfected RPMI8226 cells were collected, adjusted to a concentration of 5 × 10^7^ cells/mL, and transported on ice to the animal facility. A total of 100 μL of the cell suspension was injected subcutaneously into the right flank of each mouse using a sterile syringe. The mice were monitored every three days, and body weight and tumor size were recorded. After four weeks, the mice were euthanized, and the subcutaneous tumors were excised and photographed. Tumors were washed with saline, with a portion fixed in formalin for immunohistochemistry and the remainder stored in liquid nitrogen. For the inhibition of ATOX1 study, transfected cells and negative control cells were subcutaneously injected into the right flank of the mice. One week post-injection, the mice from each group were randomly and blindly assigned to two subgroups and received intraperitoneal injections of either DCAC50 (15 mg/kg, twice weekly) or PBS for three weeks. Mice were then sacrificed, and tumors were collected for assessment. Tumor diameters were measured every three days by an investigator who was blinded to the group assignments, and the tumor volume was calculated using the formula Volume = (*L*^2^ × *W*)/2, where *W* is the width and *L* is the length. The study was conducted in accordance with the ARRIVE guidelines. All experiments were performed in accordance with the procedures of the Institutional Animal Care and Use Committee of Huazhong University of Science and Technology, Wuhan, China. (No. 4303).

### 2.12. Mouse Tail Vein Tumor Model

Four-week-old female NSG immunodeficient mice were acclimated for two weeks. On the day of injection, cells were collected, adjusted to a concentration of 3 × 10^7^ cells/mL, and transported on ice. A total of 100 μL of the cell suspension was injected into the tail vein of each mouse using a sterile syringe. The mice were monitored regularly, and body weight was recorded. One week after injection, bioluminescence imaging was performed. The mice were anesthetized with isoflurane, and a luciferin potassium salt solution (150 mg/kg) was injected intraperitoneally. Imaging was conducted 10 min post-injection and repeated four weeks after the injection.

### 2.13. Bioinformatic Analyses

MM transcriptome sequencing data GSE6477 (15 Normal, 73 MM), GSE2658 (559 MM), GSE13591 (4 Normal, 133 MM), and GSE24080 (559 MM) and clinical information were downloaded from GEO (https://www.ncbi.nlm.nih.gov/geo/, accessed on 10th April 2024). The data were then analyzed and visualized in R 4.3.1 software. RPKM (Reads Per Kilobase of transcript per Million mapped reads) was used to normalize the sequencing data. Gene sets from MSigDB and GSEA software (http://www.gsea-msigdb.org/gsea/index.jsp, accessed on 10 April 2024) were used to conduct GSEA. The Multiple Myeloma Research Foundation (MMRF) CoMMpass (Relating Clinical Outcomes in MM to Personal Assessment of Genetic Profile) Study (NCT01454297) was downloaded from TCGA (https://portal.gdc.cancer.gov/, accessed on 10 April 2024). Single-cell sequencing data from MGUS and SMM samples can be accessed at the NCBI under accession code PRJNA694128.

### 2.14. Statistical Analysis

Analyses and statistics were performed using GraphPad Prism 9 (GraphPad Software, Boston, MA, USA, https://www.graphpad.com/, accessed on 10 April 2024). Statistical significance was evaluated using paired or unpaired *t*-tests (one- or two-tailed), depending on the sample distribution, for comparing two or more groups. One-way ANOVA followed by Tukey’s post hoc test or two-way ANOVA followed by Sidak’s post hoc test was used for grouped or multivariate analysis, and survival differences were analyzed via Kaplan–Meier curves. Data are presented as mean ± SEM, and a *p* value below 0.05 was considered statistically significant, indicated as * *p* < 0.05, ** *p* < 0.01, or *** *p* < 0.001.

## 3. Results

### 3.1. Elevated COMMD3 Expression Correlates with Poor Prognosis and Extramedullary Progression in Multiple Myeloma

**Figure 1:**
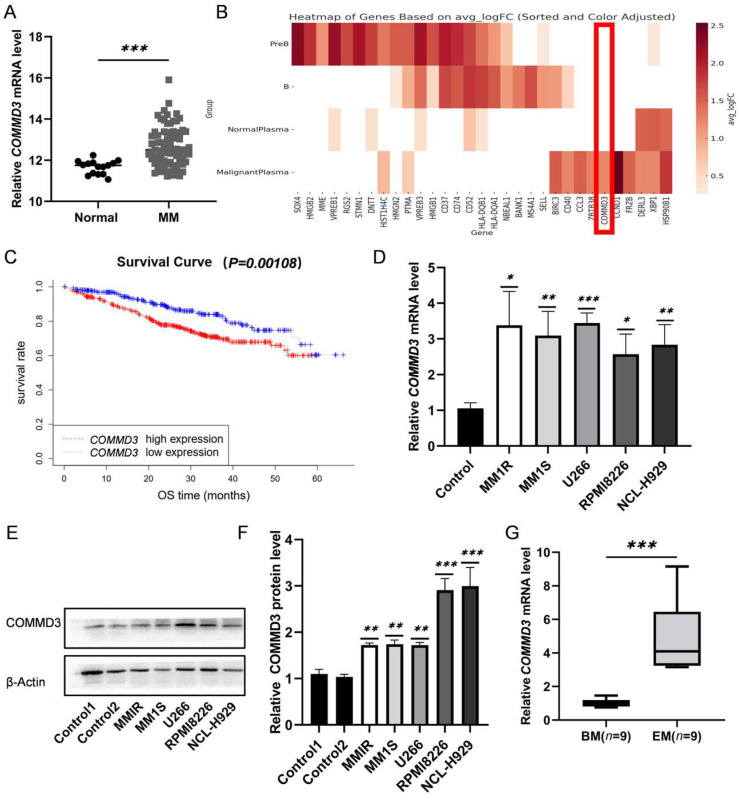
Elevated COMMD3 expression correlates with poor prognosis and extramedullary progression in multiple myeloma. (**A**) Differential expression of *COMMD3* in normal tissue (*n* = 15) vs. MM (*n* = 73) in GSE6477. (**B**) *COMMD3* expression in malignant plasma cells in single-cell sequencing data from MGUS and SMM samples. (**C**) Survival analysis from the MMRF-CoMMpass dataset demonstrated that high *COMMD3* expression was closely associated with poor prognosis in MM patients. (**D**) Relative *COMMD3* mRNA expression in MM cell lines. (**E**) Protein expression levels of COMMD3 in indicated human MM cell lines. (**F**) Relative protein expression of COMMD3 in human MM cell lines. (**G**) *COMMD3* relative mRNA levels in a panel of human BM (*n* = 9) and EM (*n* = 9) samples (* *p* < 0.05, ** *p* < 0.01, *** *p* < 0.001).

To explore the role of COMMD3 in multiple myeloma, we analyzed bulk RNA sequencing data (GSE6477), which demonstrated significant upregulation of COMMD3 in tumor samples compared to normal controls (Figure 1A). Single-cell sequencing data from the monoclonal gammopathy of undetermined significance (MGUS) to MM samples [28] also revealed a marked increase in *COMMD3* expression, particularly in malignant plasma cells (Figure 1B). A survival analysis using the MMRF-CoMMpass public database showed that elevated *COMMD3* expression was associated with shorter overall survival (Figure 1C), suggesting that COMMD3 plays a pivotal role in MM progression.

To validate these bioinformatic findings experimentally, we conducted quantitative PCR (qPCR) and Western blot analyses to examine COMMD3 expression in MM cell lines and patient-derived samples. The qPCR results confirmed significantly higher *COMMD3* mRNA levels in MM cell lines compared to normal controls (Figure 1D). Consistent with these findings, Western blot analyses demonstrated elevated COMMD3 protein expression in MM cell lines (Figure 1E,F). Furthermore, the analysis of patient-derived samples showed significantly increased *COMMD3* mRNA levels in EM tissues compared to BM samples (Figure 1G). These results highlight the potential role of COMMD3 in MM progression, particularly in extramedullary dissemination, suggesting it as both a prognostic biomarker and a potential therapeutic target.

### 3.2. COMMD3 Promotes MM Cell Proliferation and Migration

**Figure 2:**
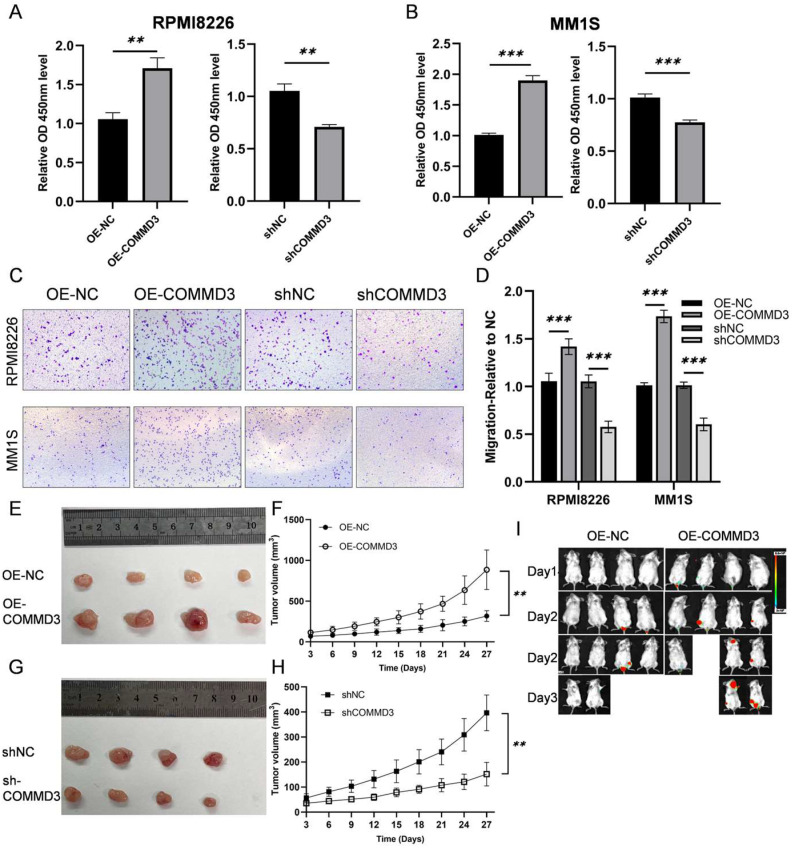
COMMD3 promotes MM cell proliferation and migration. (**A**,**B**) Cell proliferation was assessed using the CCK-8 assay in COMMD3-overexpressing cell lines (OE-COMMD3), knockdown (shCOMMD3), and their respective control groups (OE-NC and shNC). (**C**) Transwell migration assays were performed to evaluate the migration capacity of MM cells in different groups. (**D**) Quantitative analysis of metastasis frequency in the different groups. (**E**,**G**) Representative images of excised tumors from the different groups. (**F**,**H**) Tumor volume measurements in a subcutaneous xenograft model using COMMD3 overexpression (OE-COMMD3), knockdown (shCOMMD3), and their respective controls (OE-NC and shNC). (**I**) Tumor formation was evaluated in a tail vein injection model (** *p* < 0.01, *** *p* < 0.001).

Previous analyses suggested that elevated *COMMD3* expression is associated with worse overall survival in MM patients and is significantly upregulated in EM samples, implying that COMMD3 may enhance the proliferative and migratory capabilities of MM cells. To investigate this hypothesis, we generated stable COMMD3 knockdown (shCOMMD3 and shNC as negative control) and overexpression (OE-COMMD3 and OE-NC as negative control) MM cell lines via lentivirus-mediated transfection. The transfection efficiency was validated by Western blot, as shown in Supplementary Figure S1. Cell proliferation was assessed using the CCK-8 assay. The results demonstrated that COMMD3 overexpression significantly enhanced the proliferation of MM cells compared to the NC group, whereas COMMD3 knockdown resulted in a marked reduction in cell proliferation compared to the NC group (Figure 2A,B). Transwell migration assays demonstrated a similar trend. COMMD3-overexpressing cells exhibited a notable increase in migratory ability, while migration was significantly impaired in cells with COMMD3 knockdown compared to their respective controls (Figure 2C,D).

The critical role of COMMD3 in MM progression was further validated through in vivo experiments. Using a xenograft tumor model, MM cells with COMMD3 overexpression displayed significantly accelerated tumor growth compared to the control (Figure 2E,F), while COMMD3 knockdown led to a marked reduction in tumor size (Figure 2G,H). These findings provide robust evidence that COMMD3 enhances tumor proliferation in a physiological environment. Additionally, a tail vein injection model also demonstrated that COMMD3 overexpression significantly increased both tumor formation and metastasis (Figure 2I), reinforcing its role in MM progression and metastasis.

### 3.3. COMMD3 Promotes MM Proliferation and Metastasis Through Epithelial–Mesenchymal Transition and Metal Binding Regulation

**Figure 3:**
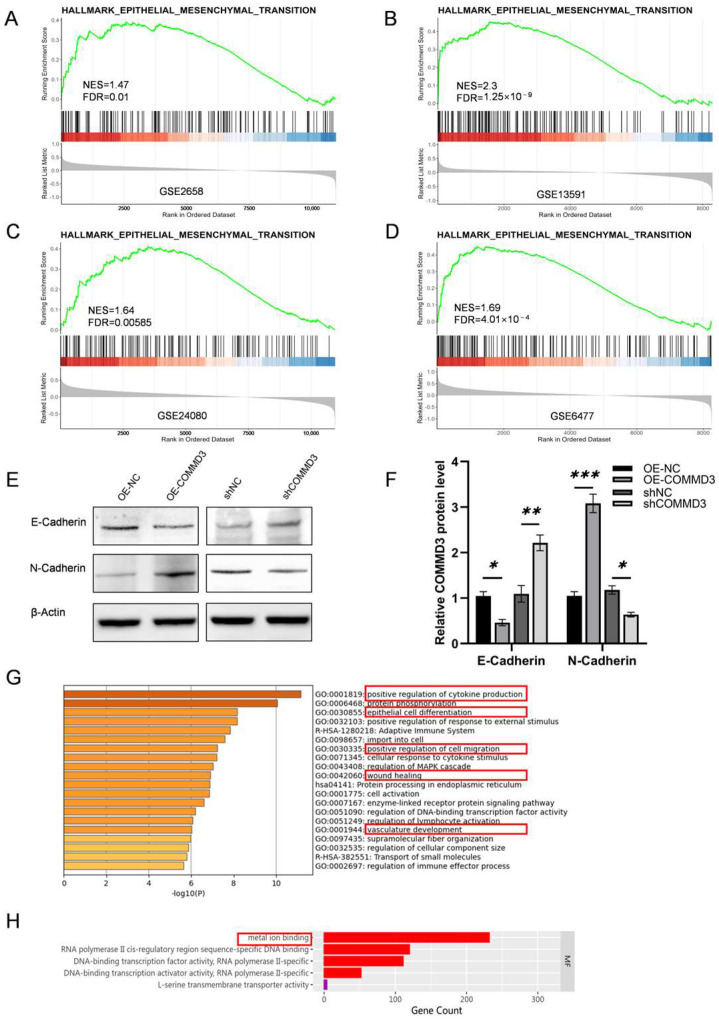
COMMD3 promotes MM proliferation and metastasis through EMT and metal binding regulation. (**A**–**D**) A GSEA analysis was conducted on differentially expressed genes between high and low COMMD3 expression groups using public MM databases (GSE2658, GSE13591, GSE24080, and GSE6477). (**E**,**F**) Western blot analysis of EMT−related protein expression in COMMD3-overexpressing cell lines (OE-COMMD3), knockdown (shCOMMD3), and their respective control groups (OE-NC and shNC). (**G**) Enrichment analysis of differentially expressed genes between COMMD3 knockdown (shCOMMD3) and control (shNC) groups in Metascape datasets. (**H**) Gene Ontology (GO) enrichment analysis of differentially expressed genes identified metal binding as a significantly enriched molecular function (MF) (* *p* < 0.05, ** *p* < 0.01, *** *p* < 0.001).

To elucidate the mechanisms underlying COMMD3-mediated proliferation and metastasis in MM, we performed a functional enrichment analysis on differentially expressed genes between groups with high and low COMMD3 expression using four publicly available databases (Supplementary Figure S2). The analysis identified key pathways related to cell proliferation and migration, such as the KRAS signaling pathway, which was activated in the high COMMD3 expression group. Notably, pathways involved in tumor metastasis, such as epithelial–mesenchymal transition (EMT), were significantly enriched (Figure 3A–D), suggesting a potential link between COMMD3 expression and metastatic progression. To validate these findings, the expression levels of EMT-associated proteins were examined in COMMD3-transfected cells. The Western blot analysis revealed a downregulation of E-cadherin and upregulation of N-cadherin (Figure 3E,F), hallmark changes indicative of EMT activation.

Transcriptome sequencing of COMMD3 knockdown and control groups provided additional insights. DEGs identified in the COMMD3 knockdown group were enriched in pathways related to tumor metastasis, including cytokine production, angiogenesis, cell migration, and EMT (Figure 3G). A Gene Ontology (GO) enrichment analysis indicated that these genes were also involved in metal ion binding processes (Figure 3G). Given the established role of COMMD family members in regulating intracellular copper ion homeostasis, these findings suggest that COMMD3 may influence MM progression through its impact on copper ion regulation.

### 3.4. COMMD3 Enhances Copper Ion Accumulation and Maintains Homeostasis in Multiple Myeloma Cells

**Figure 4:**
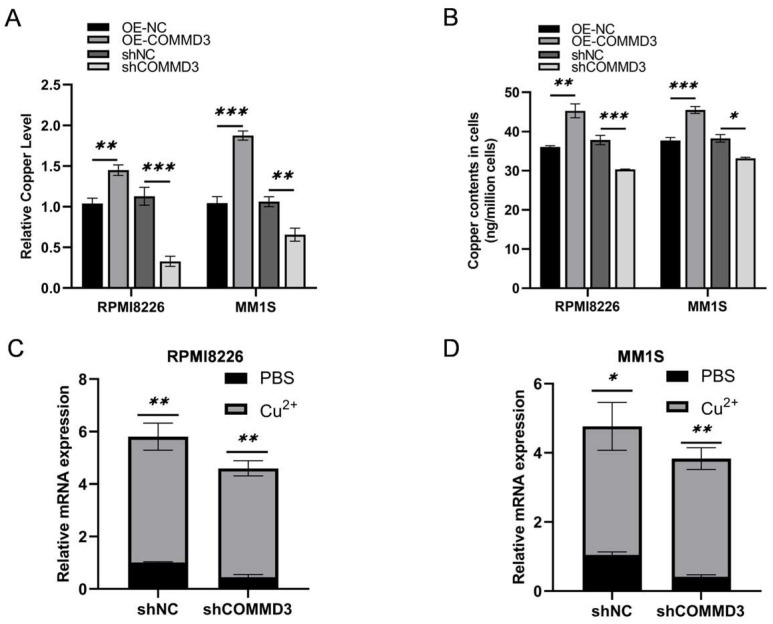
COMMD3 enhances copper ion accumulation and maintains homeostasis in multiple myeloma cells. (**A**) Copper ion levels were measured in MM cells with varying COMMD3 expression using a copper ion detection kit. (**B**) Copper ion levels were measured in MM cells with varying COMMD3 expression using inductively coupled plasma mass spectrometry (ICP-MS). (**C**,**D**) COMMD3 mRNA expression was analyzed in MM cells treated with copper compared to controls (* *p* < 0.05, ** *p* < 0.01, *** *p* < 0.001).

As a member of the copper metabolism MURR1 domain (COMMD) family, COMMD proteins have been implicated in regulating intracellular copper ion levels, with COMMD10 previously shown to influence copper metabolism in tumor cells [29]. Our transcriptomic analysis similarly indicated that COMMD3 may exert its effect through metal ion binding pathways. To validate this, we measured intracellular copper concentrations using a copper ion detection kit and inductively coupled plasma mass spectrometry (ICP-MS). The results demonstrated significantly elevated copper levels in COMMD3-overexpressing cells (OE-COMMD3 vs. OE-NC) and a marked reduction in copper levels in COMMD3 knockdown cells (shCOMMD3 vs. shNC; Figure 4A,B).

Elevated copper levels have been linked to tumor cell proliferation, angiogenesis, and the inhibition of apoptosis, thereby driving tumor progression and metastasis, while copper overload can lead to cell death [30,31,32,33]. To further investigate the relationship between copper ion concentrations and MM cells, we treated MM cells with varying concentrations of exogenous copper ions (based on previous studies [31,34,35,36]) and assessed cell proliferation and apoptosis. Interestingly, within a certain concentration range (0.1 μM, 0.5 μM, 1 μM, and 5 μM), no significant changes were observed in cell proliferation (Supplementary Figure S3A,B) or apoptosis (0.5 μM and 5 μM) (Supplementary Figure S3C,D), suggesting that MM cells possess robust mechanisms to maintain copper ion homeostasis. This ability likely promotes tumor cell survival and proliferation under conditions of copper ion stress. Furthermore, following treatment with copper ions (2.5 μM), COMMD3 expression was markedly upregulated in the COMMD3 knockdown group (Figure 4C,D), suggesting a potential compensatory response or feedback mechanism that may enhance COMMD3 expression in the presence of elevated copper ion levels.

### 3.5. The Role and Clinical Significance of ATOX1 in Multiple Myeloma

**Figure 5:**
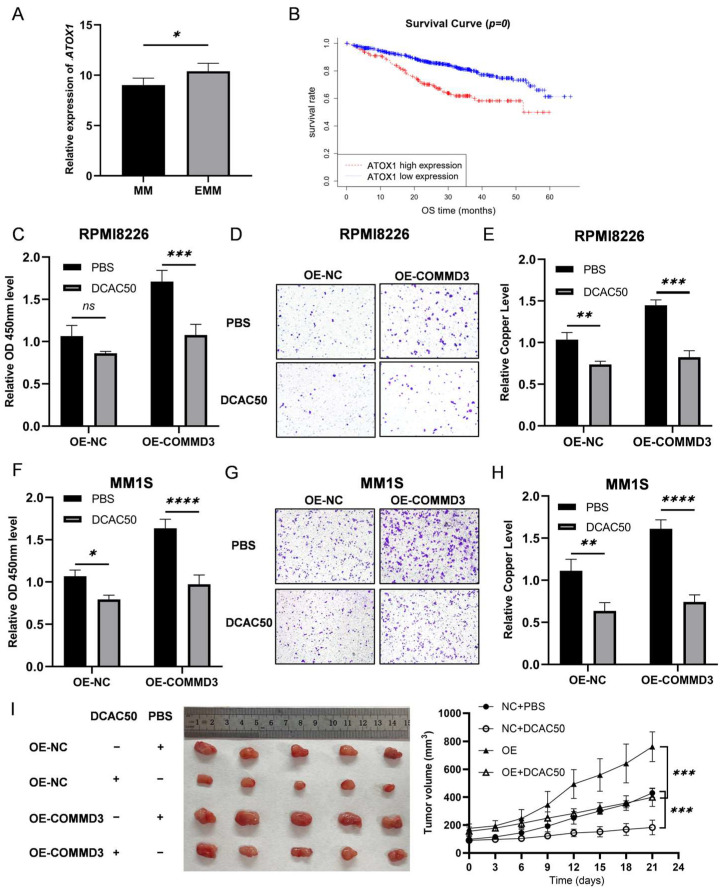
The role and clinical significance of ATOX1 in multiple myeloma. (**A**) ATOX1 expression was significantly higher in extramedullary (EM) samples compared to bone marrow (BM) samples. (**B**) Survival analysis from the MMRF-CoMMpass dataset demonstrated that high ATOX1 expression was closely associated with poor prognosis in MM patients. (**C**,**F**) CCK-8 assays were performed to assess the proliferation of OE-COMMD3 MM cells treated with the ATOX1 inhibitor DCAC50 and untreated controls. (**D**,**G**) Transwell migration assays were performed to evaluate the migration capacity of the OE-COMMD3 plus DCAC50 group compared to the OE-COMMD3 plus PBS group. (**E**,**H**) Copper ion levels were measured in MM cells with DCAC50 or PBS treatment. (**I**) Representative images of excised tumors from the DCAC50-treated groups and the control groups. The right panel is tumor volume measurements in a subcutaneous xenograft model (* *p* < 0.05, ** *p* < 0.01, *** *p* < 0.001, **** *p* < 0.0001, ns not significant).

Copper ions are significantly elevated in cancer tissues and serum, supporting tumorigenic processes through various mechanisms [11,37]. Among the proteins involved in copper metabolism, ATOX1, a copper chaperone, has been identified as a critical mediator of tumor progression. In multiple myeloma (MM), our previous transcriptomic analysis [38] revealed significantly higher *ATOX1* expression in extramedullary multiple myeloma (EMM) patients compared to those with bone-marrow-confined MM (Figure 5A). Survival analyses using the MMRF-CoMMpass public database further confirmed the clinical significance of ATOX1, as patients with elevated *ATOX1* expression exhibited significantly poorer overall survival (Figure 5B).

To further investigate the functional role of ATOX1 in MM, we employed the ATOX1 inhibitor DCAC50 to treat the cells in the COMMD3 overexpression (OE-COMMD3) groups. The CCK-8 assay measured that ATOX1 inhibition resulted in a significant reduction in MM cell proliferation (Figure 5C,F). Furthermore, the transwell migration assays revealed a marked decrease in the migratory capacity of MM cells following ATOX1 inhibition (Figure 5D,G). Copper ion levels, a functional readout of ATOX1 activity, were significantly reduced following ATOX1 inhibition, indicating its role in regulating copper homeostasis in a COMMD3-enriched environment (Figure 5E,H). In xenograft models, tumor size was markedly smaller in the DCAC50-treated group compared to the control group, confirming the role of ATOX1 in promoting tumor growth in vivo (Figure 5I).

### 3.6. COMMD3 Regulates the ATOX1-ATP7A-LOX Axis in Multiple Myeloma

**Figure 6:**
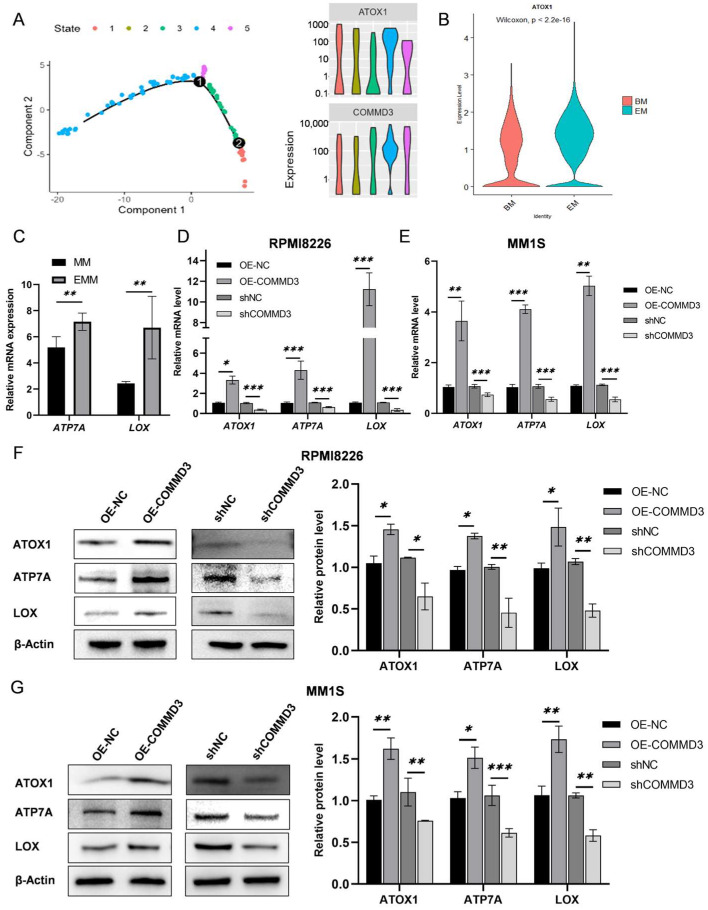
COMMD3 regulates the ATOX1−ATP7A−LOX axis in multiple myeloma. (**A**) Elevated *ATOX1* expression was observed in *COMMD3*-high-expressing myeloma cell subpopulations. (**B**) *ATOX1* expression was significantly higher in extramedullary (EM) samples compared to bone marrow (BM) samples. (**C**) *ATP7A* and *LOX* expression was significantly higher in extramedullary (EM) samples compared to bone marrow (BM) samples. (**D**,**E**) The mRNA expression levels of *ATOX1, ATP7A*, and *LOX* across groups with different COMMD3 expression levels. (**F**,**G**) The protein expression levels of ATOX1, ATP7A, and LOX across groups with different COMMD3 expression levels (* *p* < 0.05, ** *p* < 0.01, *** *p* < 0.001).

Given the pivotal role of ATOX1 in MM progression, we sought to determine whether COMMD3 regulates ATOX1 and its downstream signaling pathways. The analysis of single-cell RNA sequencing data regarding plasma cell heterogeneity revealed significantly elevated *ATOX1* expression in *COMMD3*-high MM subpopulations, with the most pronounced levels observed in extramedullary (EM) samples (Figure 6A,B). ATOX1 has been implicated in regulating the ATP7A-LOX axis, a key pathway involved in tumor cell migration and metastasis [39]. Consistent with this, we observed significantly higher expression levels of *ATP7A* and *LOX* in extramedullary multiple myeloma (EMM) patients compared to bone-marrow-confined MM patients in our previous transcriptomic analysis [38] (Figure 6C). To further investigate the regulatory relationship between COMMD3 and the ATOX1-ATP7A-LOX axis, we assessed the mRNA (Figure 6D,E) and protein levels (Figure 6F,G) of ATOX1, ATP7A, and LOX in cells with COMMD3 overexpression and knockdown. The results showed that the expression levels of these three components were significantly elevated in the overexpression group (OE-COMMD3 vs. OE-NC) and reduced in the COMMD3 knockdown group (shCOMMD3 vs. shNC). These findings indicate that COMMD3 regulates the ATOX1-ATP7A-LOX axis, potentially modulating key aspects of tumor cell behavior such as migration.

## 4. Discussion

Tumor invasion and metastasis are hallmarks of cancer [40] and major determinants of poor prognosis across various malignancies [41]. These processes involve a cascade of molecular events, including the loss of cell–cell adhesion, enhanced motility, and epithelial–mesenchymal transition (EMT), which collectively enable tumor cells to migrate and establish metastatic lesions in distant organs [42,43]. In this study, the differential gene expression analysis revealed that COMMD3 is significantly upregulated in extramedullary myeloma (EMM) samples compared to medullary counterparts. Through an integrated bioinformatics analysis and experimental validation, we demonstrated that COMMD3 plays a critical role in promoting multiple myeloma (MM) proliferation and metastasis, likely through EMT regulation and copper metabolism pathways. This highlights COMMD3 as a potential driver of EMM pathogenesis and a promising therapeutic target.

The COMMD protein family, comprising 10 members, has emerged as a key regulator of diverse cellular processes, including [42,43] tumorigenesis, metastasis, and copper metabolism [44,45,46]. Although COMMD10 is known for its role in copper–iron homeostasis and radio-resistance in hepatocellular carcinoma [29], the functional role of COMMD3 in MM has remained unexplored. Here, we show that COMMD3 enhances MM cell proliferation and migration, potentially by regulating intracellular copper ion levels. This aligns with findings in prostate cancer, where COMMD3 promotes tumor growth by driving C-MYC transcription [26]. Intriguingly, though it demonstrates context-dependent functions across tumor types, it exhibits tumor-suppressive activity in breast cancer [27] but promotes angiogenesis and tumor progression in hepatocellular carcinoma by regulating the HIF1α/VEGF/NF-κB signaling axis [20]. This tumor-specific variability underscores the complexity of COMMD3’s function and highlights its dependence on cellular context and the tumor microenvironment.

Copper dysregulation has been extensively linked to tumorigenesis, including increased angiogenesis, proliferation, and metastasis. MM cells exhibit heightened copper demand, correlating with accelerated proliferation and disease progression. Importantly, copper chelation strategies have shown efficacy in inhibiting angiogenesis and inducing apoptosis in MM [18], highlighting copper metabolism as a promising therapeutic target. Our findings further suggest that COMMD3 may act as a regulator of copper metabolism in MM. Specifically, COMMD3 expression was significantly elevated following copper ion treatment, and its knockdown led to a marked reduction in ATOX1, a copper transport protein. These observations support a potential feedback loop wherein COMMD3 regulates and is regulated by copper ion availability, amplifying its tumorigenic effects.

The involvement of the ATOX1-ATP7A-LOX axis provides further mechanistic insight into COMMD3’s function in MM. ATOX1, a key copper chaperone, has been shown to promote cell proliferation through cyclin D1 activation and facilitate cancer metastasis by accumulating at the leading edge of migrating cells [13,39]. In our study, COMMD3 knockdown reduced ATOX1 expression, and the use of an ATOX1 inhibitor after COMMD3 overexpression attenuated the effects of COMMD3, implicating COMMD3 as an upstream regulator of ATOX1-mediated pathways. This regulation may, in turn, influence ATP7A-dependent copper transport and lysyl oxidase (LOX) activity, both of which are critical for extracellular matrix remodeling and metastatic progression. Thus, the COMMD3/ATOX1-ATP7A-LOX axis emerges as a central pathway linking copper metabolism to MM proliferation and metastasis.

There are limitations regarding our work. While we demonstrate that COMMD3 regulates ATOX1 and its related pathway, it remains unclear whether COMMD3 interacts directly or indirectly with ATOX1. Further experimental studies, such as co-immunoprecipitation, proximity labeling, or structural analysis, are needed to clarify this relationship. These studies would provide a more comprehensive understanding of the underlying mechanisms. The sample sizes for the patient-derived samples are relatively small, and we look forward to increasing the sample size for further validation. There is currently a lack of small-molecule inhibitors targeting COMMD3, which could be valuable for exploring its therapeutic potential in MM.

## 5. Conclusions

In conclusion, this study suggests that COMMD3 may act as a novel regulator of MM proliferation and metastasis, with its potential tumor-promoting effects associated with the COMMD3/ATOX1-ATP7A-LOX axis and copper metabolism pathways. These findings provide insights into the molecular mechanisms underlying EMM progression and support the potential of targeting COMMD3 and copper-related pathways as therapeutic strategies. However, further studies are warranted to validate these findings in larger clinical cohorts and to elucidate the precise molecular interactions between COMMD3 and copper metabolism proteins. Additionally, future investigations should assess the therapeutic efficacy of copper chelation and COMMD3 inhibition in preclinical MM models, potentially paving the way for precision therapies aimed at preventing MM metastasis.

## Data Availability

All public data used in this study are from the open-access websites described in the Materials and Methods. The single-cell RNA sequence datasets presented in this article are not readily available because the data are part of an ongoing study. Requests to access the datasets should be directed to the corresponding author.

## Supplementary Materials

The following supporting information can be downloaded at https://www.mdpi.com/article/10.3390/biomedicines13020351/s1, Figure S1: Lentivirus transfection validation by Western blot. Figure S2: GSEA analysis of differentially expressed genes identifies key enriched pathways in high vs. low COMMD3 expression groups; Figure S3: Effects of exogenous copper ions on MM cell proliferation and apoptosis. Table S1: The primers used to assess the mRNA expression levels of genes.
