# Supplementary figures and images for: COMMD3 Regulates Copper Metabolism via the ATOX1-ATP7A-LOX Axis to Promote Multiple Myeloma Progression
Source: Biomedicines. 2025 Feb 4;13(2):351. doi: 10.3390/biomedicines13020351 (PMC11852399; doi:10.3390/biomedicines13020351)

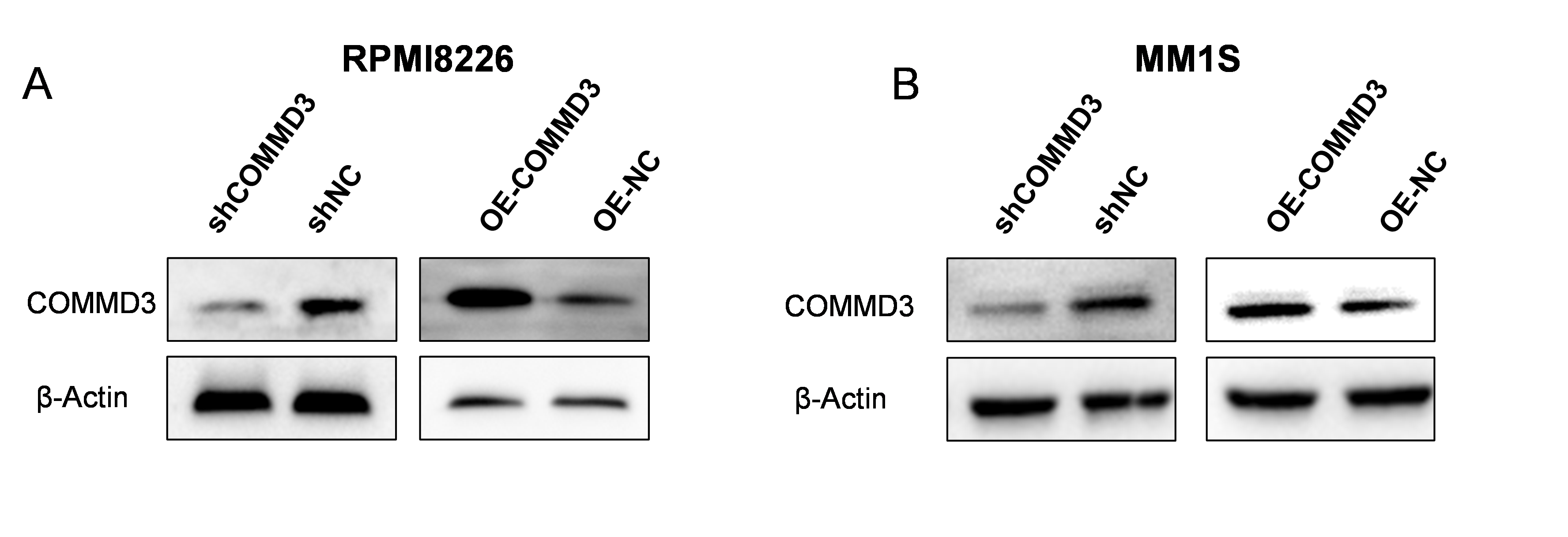

Supplement: Supplementary file 1 [file biomedicines-13-00351-s001.zip › Supplementary Figure S1.tif]

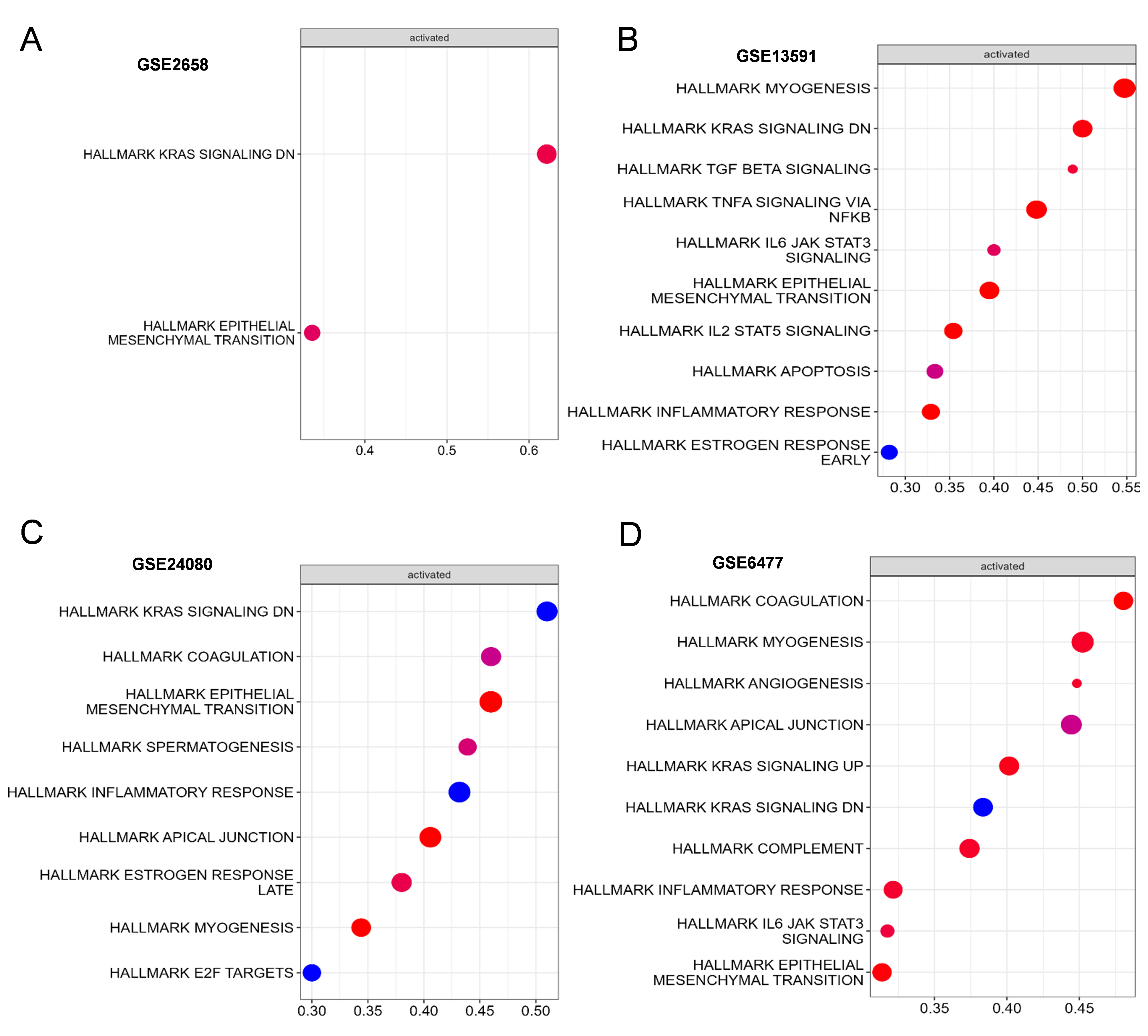

Supplement: Supplementary file 1 [file biomedicines-13-00351-s001.zip › Supplementary Figure S2.tif]

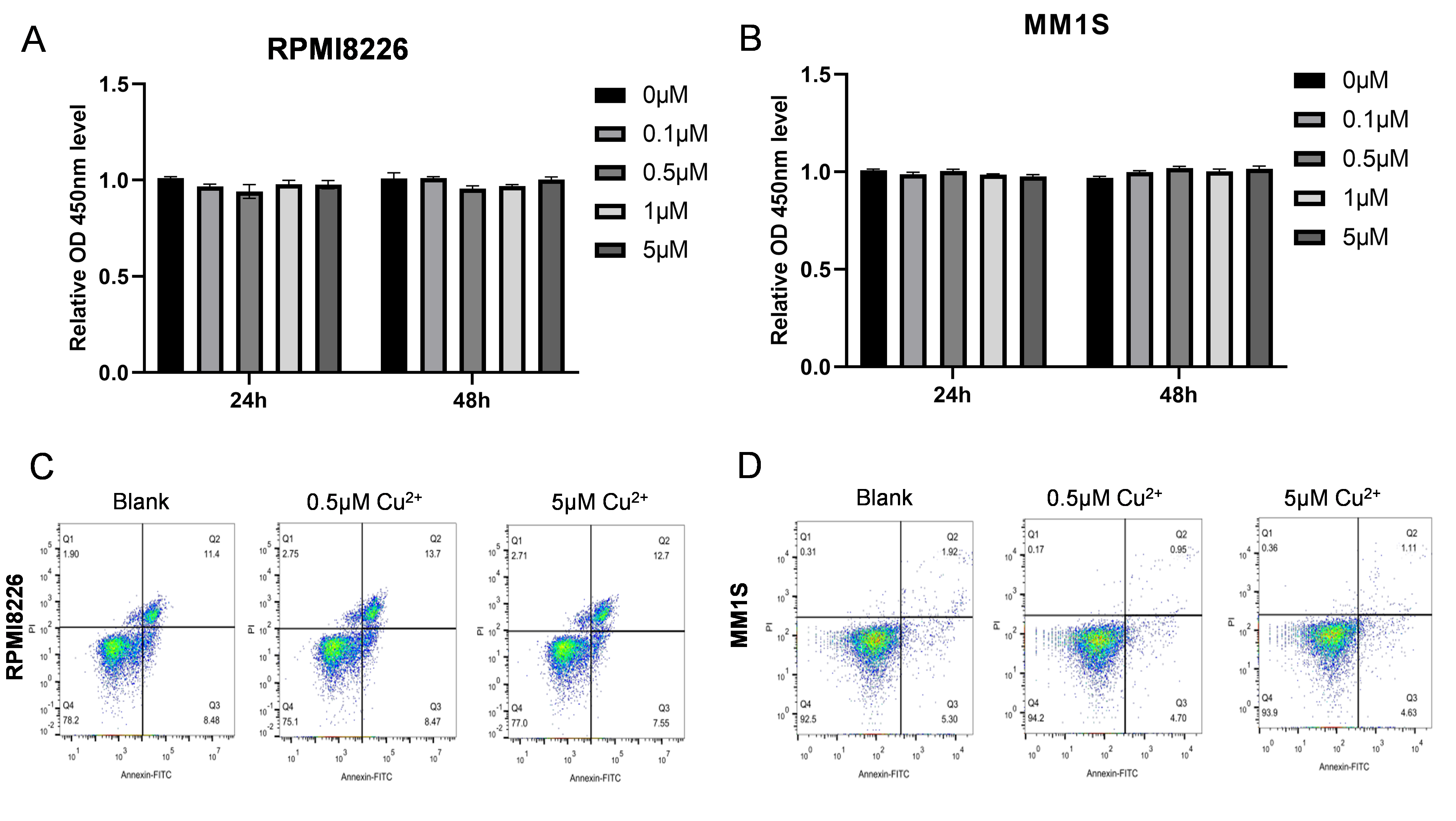

Supplement: Supplementary file 1 [file biomedicines-13-00351-s001.zip › Supplementary Figure S3.tif]
